# Genetic architecture of hippocampus subfields volumes in Alzheimer’s disease

**DOI:** 10.1111/cns.14110

**Published:** 2023-02-08

**Authors:** Jiahui Cai, Weixue Xiong, Xueqin Wang, Haizhu Tan, the Alzheimer’s Disease Neuroimaging Initiative (ADNI) Database

**Affiliations:** ^1^ Shantou University Medical College Shantou China; ^2^ Department of Statistics and Finance, School of Management University of Science and Technology of China Hefei China

**Keywords:** coimmunoprecipitation, genetic architecture, hippocampus subfields, MD simulation, molecular docking

## Abstract

**Background:**

The hippocampus is a heterogeneous structure, comprising histologically and functionally distinguishable hippocampal subfields. The volume reductions in hippocampal subfields have been demonstrated to be linked with Alzheimer's disease (AD). The aim of our study is to investigate the hippocampal subfields' genetic architecture based on the Alzheimer's Disease Neuroimaging Initiative (ADNI) data set.

**Methods:**

After preprocessing the downloaded genetic variants and imaging data from the ADNI database, a co‐sparse reduced rank regression model was applied to analyze the genetic architecture of hippocampal subfields volumes. Homology modeling, docking, molecular dynamics simulations, and Co‐IP experiments for protein–protein interactions were used to verify the function of target protein on hippocampal subfields successively. After that, the association analysis between the candidated genes on the hippocampal subfields volume and clinical scales were performed.

**Results:**

The results of the association analysis revealed five unique genetic variants (e.g., ubiquitin‐specific protease 10 [*USP10*]) changed in nine hippocampal subfields (e.g., the granule cell and molecular layer of the dentate gyrus [GC‐ML‐DG]). Among five genetic variants, USP10 had the strongest interaction effect with BACE1, which affected hippocampal subfields verified by MD and Co‐IP experiments. The results of association analysis between the candidated genes on the hippocampal subfields volume and clinical scales showed that candidated genes influenced the volume and function of hippocampal subfields.

**Conclusions:**

Current evidence suggests that hippocampal subfields have partly distinct genetic architecture and may improve the sensitivity of the detection of AD.

## Graphical abstract

We downloaded the imaging data, clinical data, and genetic data from the Alzheimer's Disease Neuroimaging Initiative (ADNI; http://adni.loni.usc.edu/). After extracting the hippocampal subfields of the download imaging data by the FreeSurfer software (version 6.0) 27 and selecting the nonsynonymous variants, SNPS are more sensitive to hippocampal subfields by using GWAS analysis. Co‐sparse reduced rank regression (CSRRR) and simple linear regressions were used to analyze the association between the 12 hippocampal subfields and the selected nonsynonymous variants. Finally, we also combined experimental methods (coimmunoprecipitation (Co‐IP)) and computational methods (homology modeling, molecular docking, and molecular dynamics (MD) simulation) to reveal the biological mechanism of effector genes involved in AD.

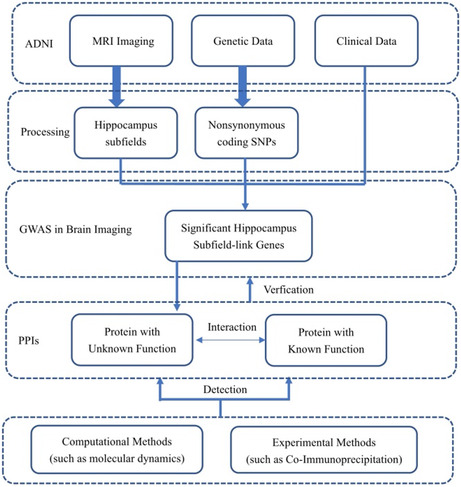

## 1 INTRODUCTION

The hippocampus plays an important role in learning, memory, and spatial navigation.^1^ It implicates several brain disorders, especially Alzheimer's disease (AD). Previous studies have shown that the hippocampus is particularly vulnerable to pathological conditions.^2^ For example, cell loss and neuropathologic changes (including intraneuronal neurofibrillary tangles (NFTs) containing hyperphosphorylated tau protein, deposition of Aβ protein, and extensive neurodegeneration) are first found in some brain regions with asymmetric and progressive atrophy, like the dorsal raphe nucleus (DRN) in the brainstem,^3^ and medial temporal lobes(MTL).^4^, ^5^, ^6^, ^7^, ^8^ The atrophy is believed to be associated with functional deficits in AD.^9^ Hippocampal atrophy, determined by magnetic resonance imaging (MRI),^10^ is considered as one of the most validated, easily accessible biomarker of AD and has been widely used.^11^

The hippocampus is composed of several subfields with different histological characteristics and heterogenous structure.^12^ It includes the cornu ammonis (CA1‐CA4) and the dentate gyrus (DG),^13^ and for differentiated prodromal AD, preliminary findings give evidence that estimates of the volume of hippocampal subfields are more sensitive than that of the total hippocampal volume.^14^, ^15^ Pathologically, NFTs are investigated in CA1, subiculum, CA2, CA3, and CA4/DG in patients with mild cognitive impairment (MCI).^16^ Aβ precedes NFTs formation.^8^ Aβ is detected extra‐ and intracellularly, whereas NTFs are found to be located intracellularly within Aβ‐containing neurons in the CA1 of AD mouse.^8^ In addition, a large amount of research in hippocampus has identified that the volume reductions in hippocampal subfields such as CA1, subiculum, and dentate gyrus(DG) have been demonstrated to be linked with AD.^2^, ^17^, ^18^, ^19^, ^20^ For instance, the CA1is anatomical, physiological, and functional heterogeneities in the proximal‐distal, dorsal‐ventral, and anterior–posterior axes of hippocampus.^21^, ^22^ These studies demonstrate that the hippocampal subfields with unique properites and differential vulnerability to some neuropsychiatric diseases, which are considered as sensitive biomarkers in the early AD detection.

Imaging genetic studies confirm that hippocampal volume is a highly polygenic trait.^2^, ^23^ As the development of the emergence of high field MRI scanners and more sophisticated neuroimaging methods introduced,^24^ the genetic architecture, the lifespan changes in hippocampal subfields volumes, and the functions of them are investigated.^25^, ^26^ Wang et al. show that a cognitively normal elderly population that carriers of the *TREML2* gene have larger volumes of CA1 by using multiple linear regression.^25^ Furthermore, Ambrée et al. reported that the number of proliferative cells in the DG decreases in H1R knockout mice, which have deficits in spatial learning and memory.^27^

The first hypothesis of our study is that the changes in the different volumes of hippocampal subfields have the different genetic architecture because the discrepancy of the cytoarchitecture, connectivity patterns, and functions are existed in the hippocampal subfields.

Protein–protein interactions (PPIs) are established to construct metabolic and signal pathways to get function because dysfunctions and malfunction of pathways and alterations in PPIs have shown to be related to some diseases, like neurodegenerative disease^28^ (such as AD).^29^ The β‐secretase enzyme, β‐site amyloid precursor protein‐cleaving enzyme 1 (BACE1) is known to be associated with AD by hydrolysing amyloid precursor protein (APP) to produce Aβ.^30^, ^31^ BACE1 cleaves APP in the first step in β‐amyloid (Aβ) peptide production. PPIs between nuclear factor kappa‐B (NF‐κB) interaction with BACE1 enhances BACE1 transactivation and promotes amyloid production in AD.^32^, ^33^ The regulations of BACE1 are also related to AD.^34^ For example, BACE1 accumulation in axonal swellings is triggered by GGA3, which is linked to late‐onset AD.^34^ BACE1 exhibits prominent localization in the stratum lucidum of the hippocampus, composed of axons and presynaptic terminals of mossy fibers from granule cells in the dentate gyrus.^35^ Local elevation in BACE1 processing could contribute to amyloid burden in the progress of AD.^36^ To determine the potential molecular structure‐to‐function of the candidate proteins in AD, atomic‐molecular dynamics (MD) simulation, and co‐immunoprecipitation (Co‐IP) reveal the complete microscopic model of PPIs and determine the potential molecular structure‐to‐function of the candidate proteins in AD. Hence, the second hypothesis is that candidate proteins may be involved in BACE1 regulation in AD through PPIs which can be verified by MD simulation and Co‐IP in our study.

According to these two hypotheses, after downloading the imaging data, clinical data and genetic data from the Alzheimer's Disease Neuroimaging Initiative (ADNI; http://adni.loni.usc.edu/), we extracted the hippocampal subfields by the Freesufer software (version 6.0)^37^ and selected the coding nonsynonymous variants, constitute more than 50% of the mutations known to be involved in human inherited diseases,^38^ by filtering pipelines. Co‐sparse reduced rank regression (CSRRR) and simple linear regressions were used to analyze the association between the 12 hippocampal subfields of two hemispheres and the selected non‐synonymous variants. Finally, we also combined experimental methods (co‐immunoprecipitation (Co‐IP)) and computational methods (homology modeling, molecular docking, and molecular dynamics (MD) simulation) to reveal the biological mechanism of effector genes involved in AD.

## 2 MATERIALS AND METHODS

**FIGURE 1:**
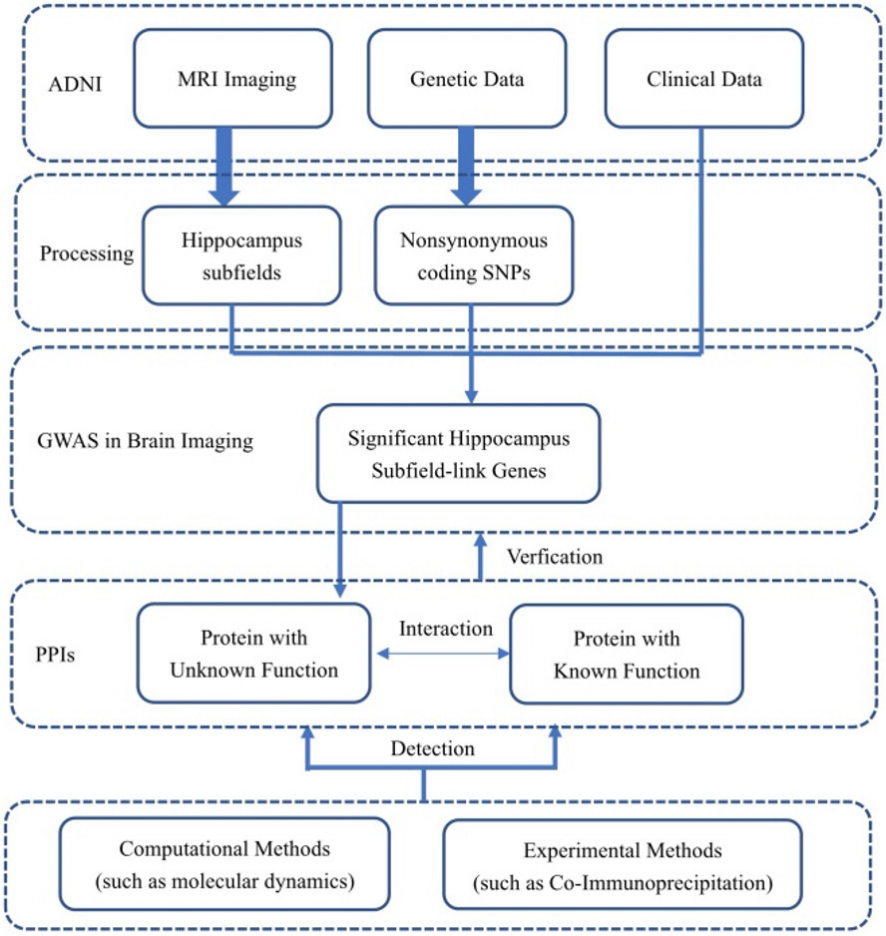
Analysis Workflow.

Detailed procedures for the association analysis between imaging phenotypes and genetic variants are provided in Figure 1.

### 2.1 ADNI database

In our study, approximately 600,470 variants on chromosome 1–22 and 1.5 T accelerated T1‐weighted structural MRI scans of primarily the hippocampus in 175 AD and 214 NC individuals were acquired from the ADNI‐1 database(http://adni.loni.usc.edu/). Demographic and clinical data (e.g., age, gender, *APOE4*, the Mini‐Mental State Examination (MMSE) scale, and Geriatric Depression Scale (GDS)) were also gathered. Selection and exclusion criteria are available on the ADNI website (http://adni.loni.usc.edu/wp‐content/uploads/2010/09/ADNI_GeneralProceduresManual.pdf). The investigators within ADNI did not participate in analysis or writing of this manuscript. Information about written Informed or phone consent, all relevant ethical guidelines and/or ethics committee approvals were seen in the ADNI‐1 data set.

## 3 PREPROCESSED DATA

### 3.1 Participants

The normal distribution was tested by the two‐sample of Kolmogorov–Smirnov test, and the homogeneity of variance was also checked by Levene's Test. The results of variance inflation factor (VIF) and minimum covariance determinant (MCD) were applied to handle collinearity and mis‐measured outliers in our study. To compare the difference of demographic characteristics and 12 hippocampal subfields between two groups, several two‐sided parametric or nonparametric difference analyses were performed due to the distribution of the data.

### 3.2 FreeSurfer‐based segmentation of hippocampal subfields

The FreeSurfer software was applied to analyze hippocampal subfields volumes in patients with AD along with data from matched controls in our study because of its automation, availability, and higher accuracy.^39^ The hippocampal subfield segmentation was based on a Bayesian modeling approach and manual delineations of each hippocampal subfield by FreeSurfer.^40^, ^41^ The outputs of the hippocampal segmentation are left and right hemisphere images with label assignments for voxels in the hippocampal area to one of twelve subregions^42^: CA1, molecular layer (ML), hippocampal tail, subiculum, presubiculum, granule cell layer of dentate gyrus (GC‐ML‐DG), CA4, CA3, hippocampal fissure, hippocampus‐amygdala‐transition‐area (HATA), and fimbria. After hippocampus segmentation, FreeSurfer was applied to obtain volumes of the hippocampal subfields, total hippocampal formation volume, and intracranial volume^40^ in our study.

The procedures of the segmentation of hippocampal subfields were fully automated without manual editing. All the images were checked and interpreted by 1 psychiatric resident physician and 1 radiologist. One subject was excluded because of the poor image quality.

### 3.3 Selection on nonsynonymous mutations

Nonsynonymous mutations change the sequence of amino acids and then affect the genetic function, while the synonymous mutations do not affect the genetic function.^43^ Nonsynonymous mutations were obtained by filtering according to the following pipeline: quality control (QC) was carried out using the PLINK software 1.90 beta (developed by Christopher Chang with support from the NIH‐NIDDK's Laboratory of Biological Modeling, the Purcell Lab, and others); genetic imputation was performed on the Michigan imputation server (https://imputationserver.sph.umich.edu/index.html#!pages/home), which was a new web‐based service for imputation that facilitated access to new reference panels; and then, annotation was carried out to determine nonsynonymous variants by using the ANNOVAR software, an efficient software tool to utilize update‐to‐date information to functionally annotate genetic variants detected from diverse genomes (http://www.openbioinformatics.org/annovar/),^44^, ^45^ since non‐synonymous variants were impacted by the degree of genetic diversity and pattern of linkage disequilibrium^46^; principal components (PCs) analysis was subsequently done using the EIGENSTRAT, a leading association mapping method in terms of its popularity, power, and type I error control.

### 3.4 Association analysis between imaging phenotypes and genetic variants

Age, sex, and APOliprotein E4(*APOE4*) are the great risk factors for Alzheimer's disease.^47^, ^48^, ^49^ To eliminate the influence of covarites (an intercept, age, sex, intracranial volume,^50^ *APOE4*, and several top significant PCs in SNPs), we regressed hippocampal subfields to these covariates. The resulting residuals and the selected 11,596 nonsynonymous variants were treated as response (Y, 24 hippocampal subfields because of 12 hippocampal subfields for per hemisphere) and explanatory variables (X), respectively. CSRRR^51^ was then performed because it is an efficient way to select causal nonsynonymous variants and affected hippocampal subfields simultaneously via nonconvex penalty based on a group primal dual‐active set formulation. The following formula depicted the CSRRR model used in our study:

$$\min_{C}{\left|\left|Y-\mathrm{XC}\right|\right|}_{F}^{2},\text{subject to}\;\mathrm{rank}\left(C\right)\leq r,{\left|\left|C\right|\right|}_{2,0}=k_{x},{\left|\left|C^{T}\right|\right|}_{2,0}=k_{y},$$

where C denoted the coefficient matrix linking the volumes for the 24 hippocampal subfields. C∈ℝ^p × q^ (ℝ^p^ represents p‐dimensional (*p* = 11,596) genetic variation, ℝ^q^ represents q‐dimensional (*q* = 24) image phenotype), ${\left|\left|∙\right|\right|}_{F}$ denoted the Frobenius norm, rank(∙) indicated the matrix rank, and ${\left|\left|C\right|\right|}_{2,0}$ counted the number of nonzero rows in C, 1 $\leq r\leq \min\left(\mathrm{rank}\left(X\right),q,k_{x},k_{y}\right)$, 1 $\leq k_{x}\leq \min\left(p,n\right)$, and 1 $\leq k_{y}\leq \min\left(q,n\right)$. K_x_ represented the desired levels of sparsity in genotypes, k_y_ the desired levels of sparsity in phenotypes, and *r* represented the rank of the coefficient matrix.^51^

Based on the results of CSRRR analysis, the minimum *p*‐value of each SNP was the smallest *p*‐value by regressing the SNP to each selected hippocampal subfields. Bonferroni correction was applied to correct for multiple comparisons.

In order to analyze the effect of selected SNPs on hippocampal subfields function, image phenotype, and clinical scales, statistical analysis of hippocampal subfields was performed using Student's *t*‐test, Wilcoxon rank‐sum test, or chi‐square test due to the disease grouping and variation grouping information.

## 4 EXPERIMENTAL VALIDATION OF COMPUTATIONAL BIOLOGY AND CELL BIOLOGY

### 4.1 Docking and MD simulation of the selected SNPs with BACE1 complex

Docking and MD simulations were introduced to verify the stability of the resulting AD complex and validate the pathogenesis involved in AD.^52^, ^53^, ^54^ Several published proteins related to AD can be used as receptors in the docking and MD simulation, such as the α secret enzyme, γ secret enzyme, and BACE1.^55^, ^56^, ^57^ BACE1 is a promising target for the treatment of AD^57^ and was chosen to dock with significant nonsynonymous variants in our study because AD is characterized by Aβ, which is generated by BACE1.^30^, ^58^, ^59^, ^60^ Therefore, proteins that were transcribed by the significant nonsynonymous variants were modeled in three different ways, respectively (including the SWISS‐MODEL, the first fully automated protein homology modeling server in comparative modeling; the I‐TASSER server, which has recently been ranked as the best server for protein structure prediction community wide^61^; and the AlphaFold Protein Structure Database (AlphaFold DB, https://alphafold.ebi.ac.uk), an openly accessible and extensive database of high‐accuracy protein structure predictions powered by AlphaFold v2.0 of DeepMind). The point amino acids were then mutated using Chimera 1.14, which is a program for the interactive visualization and analysis of molecular structures and related data.^62^ The qualities of the protein structures were evaluated using PROSA, which is a suite of programs to check the stereochemical quality of protein structures.^63^ Each wild and mutant protein was subsequently docked with BACE1 by HADDOCK,^64^ and MD simulations were performed.

### 4.2 Co‐IP experiment

To verify whether the increase of Aβ deposition would be induced by PPIs between the candidated proteins and BACE1 which caused the hippocampal atrophy in AD patients, we conducted a Co‐IP experiment based on the results of MD simulation. Human embryonic kidney 293 cells (HEK 293 T) carrying SV40 large T antigen (Cat: CRL‐11268), not human tissues, obtained from the ATCC and used in the Co‐IP experiment. We first cotransfected pCDAN3.1(+)‐Flag‐BACE1 and pCDAN3.1(+)‐HA‐*USP10* plasmids into HEK 293 T cells for 48 hours. Then, HEK 293 T cells were subjected to a Co‐IP assay using anti‐Flag magnetic beads. Anti‐HA magnetic beads were coimmunoprecipitated with cotransfected HEK 293 T cells. Finally, western blotting was conducted using Flag rabbit polyclonal antibody. Detailed information on the Co‐IP experiment is available in the supplementary materials.

### 4.3 The association analysis between the candidated genes on the hippocampal subfields volume and clinical scales

To confirm the effect of candidate genes on the volume of hippocampal subfields and clinical scale, the Mini‐Mental State Examination (MMSE) and the Geriatric Depression Scale (GDS) scales were downloaded from the ADNI database. The MMSE consisted of 10 items including testing orientation, memory, attention, calculation, language, and visual–spatial ability. The GDS composed of 15 questions and was designed to identify symptoms of depression in the elderly. Participants with valid scale scores were included in the analyses. Several two‐sided parametric or nonparametric difference analyses were performed to compare the difference of 24 hippocampal subfields volumes and the score of scales between two groups (homozygous variants and heterozygous variants) based on the distribution of the data.

A *p*‐Value <0.05 was considered statistically significant in some analyses. All the statistical analyses were carried out using the R (version 4.2.0).

## 5 RESULTS

### 5.1 Preprocessed data

After a series of preprocessing steps on the genetic data (see Supplementary Materials and Methods for details), a total of 11,596 SNPs in 150 AD patients and 180 normal controls (NCs) were reserved as the independent variables. Six top significant PCs were treated as additional covariates. After hippocampal segmentation, 24 hippocampal‐subfields volumes (continuous data) were extracted as the high‐deminsional dependent variables. The results of VIF indicated that there was no collinearity between variables. No outlier was existed according to the results of MCD(see Supplementary Materials Table S2). The results of difference analysis between the AD and HC cohorts showed that no differences in age (*p*‐value = 0.907), sex (*p*‐value = 0.815), and race (*p*‐value = 0.357) were existed (see Supplementary Materials Table S3). APOE4, an important genetic biomarker for AD pathophysiology, was significantly different between the AD and HC (*p*‐value < 0.001) (see Supplementary Materials Table S3).

### 5.2 Association analysis between imaging phenotypes and genetic variants

**FIGURE 2:**
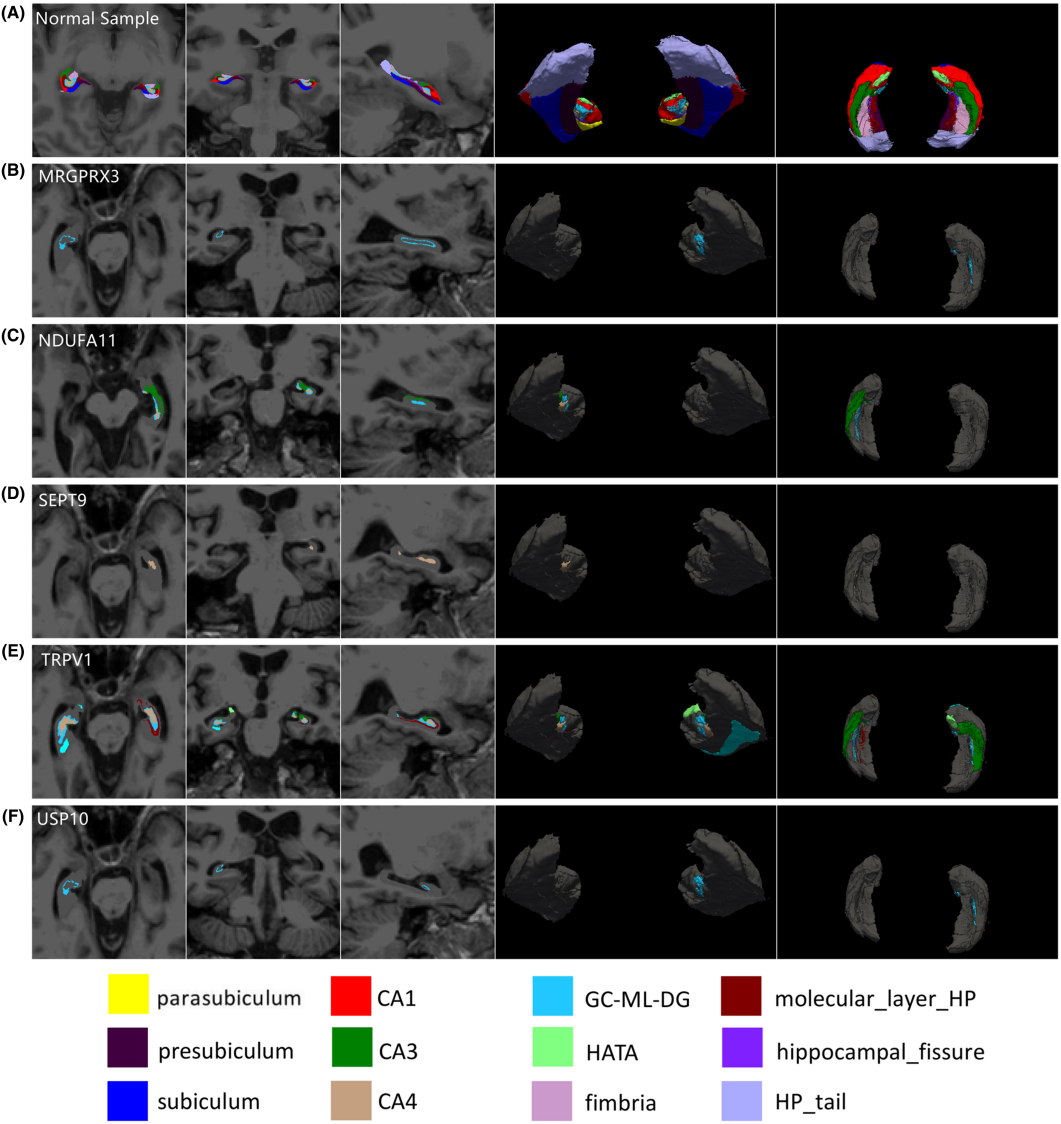
Association of Significant Genes with Hippocampal Subfields. (A): each hippocampal subfields of Normal Sample. (B): MRGPRX3 was associated with right GC‐ML‐DG and left CA4. (C): NDUFA11 was associated with left GC‐ML‐DG and left CA3. (D): SEPT9 was associated with left CA4, left GC‐ML‐DG, right GC‐ML‐DG, right CA4 and right CA3. (E): TRPV1 was associated with left CA4, right HATA, left molecular layer, right subiculum and left CA3. (F): USP10 was associated with right GC‐ML‐DG. From left to right column: Axial, Coronal, Sagiital, Posterior 3D render, Superior 3D render.

By applying CSRRR and linear regression, we identified five genetic variants associated with the hippocampal subfields. Table 1 described the variant in detail. Figure 2 depicted the genetically affected hippocampal subfields on standard resolution magnetic resonance imaging (MRI). The significance threshold was set at 0.05/number of independent variants.

**TABLE 1:** The results of the association analysis between imaging phenotypes and genetic variants.

Gene name	Hippocampal subfield	Estimate	Position	rs number	*p*_value_ *	*p*_adjust_ *
*MRGPRX3*	Right GC‐ML‐DG	21.11831619	11:18159668:T:C	rs79562368	0.0022	0.0460
	Right GC‐ML‐DG	21.11831619	11:18159669:G:A	rs78408237	0.0022	0.0460
*NDUFA11*	Left CA4	17.4615071	19:5893058:G:A	rs12980262	0.0007	0.0144
	Left GC‐ML‐DG	20.35604934	19:5893058:G:A	rs12980262	0.0008	0.0165
	Left CA3	15.04546368	19:5893058:G:A	rs12980262	0.0014	0.0299
*SEPT9*	Left CA4	−9.20776111	17:75401190:G:A	rs2164449	0.0024	0.0495
*TRPV1*	Left GC‐ML‐DG	15.20083846	17:3486702:G:A	rs224534	0.0001	0.0021
	Right GC‐ML‐DG	15.74644245	17:3486702:G:A	rs224534	0.0002	0.0034
	Right CA4	13.11314151	17:3486702:G:A	rs224534	0.0002	0.0041
	Right CA3	12.592699	17:3486702:G:A	rs224534	0.0002	0.0048
	Left CA4	12.2399324	17:3486702:G:A	rs224534	0.0002	0.0048
	Right HATA	3.890031124	17:3486702:G:A	rs224534	0.0004	0.0078
	Left molecular layer	25.83695343	17:3486702:G:A	rs224534	0.0010	0.0208
	Right subiculum	21.73799455	17:3486702:G:A	rs224534	0.0014	0.0285
	Left CA3	9.651440631	17:3486702:G:A	rs224534	0.0016	0.0327
*USP10*	Right GC‐ML‐DG	−15.20300583	16:84778694:T:C	rs2326391	0.0021	0.0448
	Right GC‐ML‐DG	−15.20300583	16:84778697:G:C	rs1812061	0.0021	0.0448

* As shown, *p*‐values are not corrected for multiple comparisons, and *p*‐value_adjust_ are corrected after Bonferroni correction.

## 6 EXPERIMENTAL VALIDATION OF COMPUTATIONAL BIOLOGY AND CELL BIOLOGY

### 6.1 Docking and MD simulation of the selected SNPs with BACE1 complex

As shown in Table 2, USP10 had the strongest interaction with BACE1 in these selected SNPs. The interaction of the BACE1‐ubiquitin‐specific protease 10 (USP10) complex was weaker than that of the mutant BACE1‐USP10 Val204Leu (rs1812061). The high ambiguity driven protein–protein docking (HADDOCK) score of BACE1‐USP10 Val204Leu (−63.0 ± 17.5) was the lowest, which suggested that its interaction was the highest. We also found that the proteins MRGPRX3 and TRPV1 did not interact with BACE1 because their HADDOCK score >0. All three modeling modalities showed that USP10 interacted with BACE1 (HADDOCK score <0).

**TABLE 2:** Docking results of different proteins with BACE1 by different modeling software.

	SWISS‐MODEL					I‐TASSER		AlphaFold DB
	MRGPRX3_BACE1	NDUFA11_BACE1	SEPT9_BACE1	TRPV1_BACE1	BACE1_USP10	BACE1_USP10	BACE1_USP10 Val204Leu	BACE1_USP10
HADDOCK score	84.0 ± 16.1	−37.3 ± 9.8	−17.9 ± 6.8	127.8 ± 12.2	−77.5 ± 1.6	−59.1 ± 7.9	−63.0 ± 17.5	−22.0 ± 2.0
Cluster size	8	7	52	31	14	10	18	30
RMSD from the overall lowest‐energy structure	0.5 ± 0.3	0.5 ± 0.3	17.6 ± 0.0	8.6 ± 0.2	0.5 ± 0.3	0.7 ± 0.5	0.4 ± 0.3	3.3 ± 0.2
Van der Waals energy	−127.0 ± 1.6	−82.0 ± 11.3	−71.5 ± 8.6	−114.2 ± 7.0	−57.0 ± 2.3	−83.2 ± 0.7	−85.9 ± 4.3	−73.0 ± 1.9
Electrostatic energy	−132.3 ± 19.7	−393.7 ± 54.1	−272.2 ± 30.9	−204.2 ± 40.9	−555.9 ± 42.5	−453.5 ± 46.7	−574.7 ± 72.9	−312.3 ± 48.1
Desolvation energy	−64.0 ± 4.2	−28.1 ± 2.8	0.6 ± 4.9	−30.2 ± 4.8	2.5 ± 2.1	2.8 ± 3.3	4.6 ± 3.6	9.8 ± 3.3
Restraints violation energy	3014.3 ± 140.9	1515.0 ± 71.2	1074.3 ± 157.1	3130.2 ± 142.3	881.6 ± 66.6	1119.6 ± 71.7	1332.9 ± 154.7	1036.1 ± 55.5
Buried Surface Area	4162.9 ± 84.7	3393.2 ± 55.7	2776.3 ± 177.3	3474.1 ± 77.4	2597.5 ± 23.9	3117.9 ± 105.3	3389.9 ± 216.2	2731.6 ± 103.1
Z‐Score	−1.8	−1.8	−1.2	−1.1	−2.4	−1.7	−2.5	−1.4

*Note*: 1. SWISS‐MODEL(https://swissmodel.expasy.org) was the first fully automated protein homology modeling server in comparative modeling. 2. The iterative threading assembly refinement (I‐TASSER) server is an integrated platform for automated protein structure and function prediction based on the ab initio folding. 3. The AlphaFold Protein Structure Database (AlphaFold DB, https://alphafold.ebi.ac.uk) is an openly accessible, extensive database of high‐accuracy protein‐structure predictions, powered by AlphaFold v2.0 of DeepMind. 4. The HADDOCK score was obtained by the weighted average of the Van der Waals, electrostatic, and desolvation energies, and the buried surface area represents the interaction strength of the protein complex binds.

**FIGURE 3:**
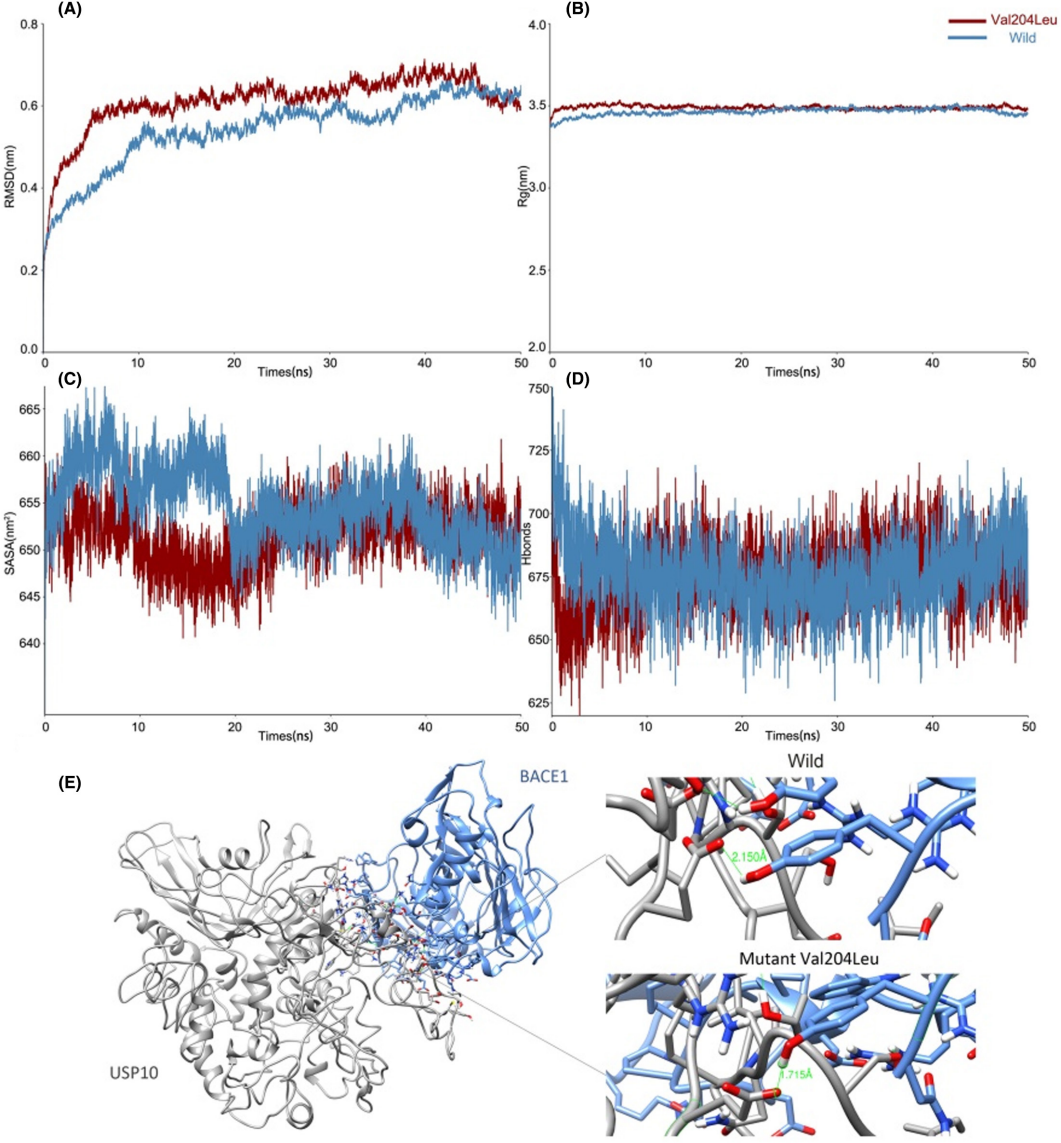
MD simulation results of BACE1‐USP10 and BACE1‐USP10 Val204Leu. The blue line represents the wild type and the red line represents the mutant BACE1‐USP10 Val204Leu. (A). The root mean square deviation (RMSD) plot shows that there were no erratic fluctuations in the molecular systems, and all complexes were stable. (B). The results of the radius of gyration (Rg) show the volumetric and compactness variation induced by the complex. (C). The results of the solvent accessible surface area (SASA) for the protein structures show the dimensional discrepancy. (D). The results of the hydrogen bonds account for protein rigidity and the protein's ability to interact with its partners. (E). The distance of the intermolecular hydrogen bond between the 384 tyrosine (Tyr) of mutant USP10 Val204Leu and 62 glutamic acid (Glu) of BACE1 was closer than that of wild USP10.

Docking and MD simulations showed that no erratic fluctuations existed in the molecular systems and all the complexes were stable according to Figure 3A. Figure 3B displays that the volumetric and compactness variations were induced by the complex, suggesting the system became more tight and stable after mutation.^65^ The solvent accessible surface areas (SASA) for the protein structures show the dimensional discrepancy in 50 ns between the wild type and its mutants.^66^ Hence, the SASA of the mutant BACE1‐USP10 Val204Leu was smaller than that of the wild type, indicating the interaction contact area of its complex was smaller (see Figure 3C). From Figure 3D, the number of hydrogen bonds accounting for protein rigidity and the protein's ability to interact with its partners in BACE1‐USP10 Val204Leu were lower than in wild type. As Figure 3E showed, the distance of the intermolecular hydrogen bond between the 384 tyrosine (Tyr) of mutant USP10 Val204Leu and 62 glutamic acid (Glu) of BACE1 was closer than that of wild USP10. Consequently, the reason for the enhanced interaction of the mutants may be caused by the shortened length of hydrogen bonds.

### 6.2 Co‐IP experiments

**FIGURE 4:**
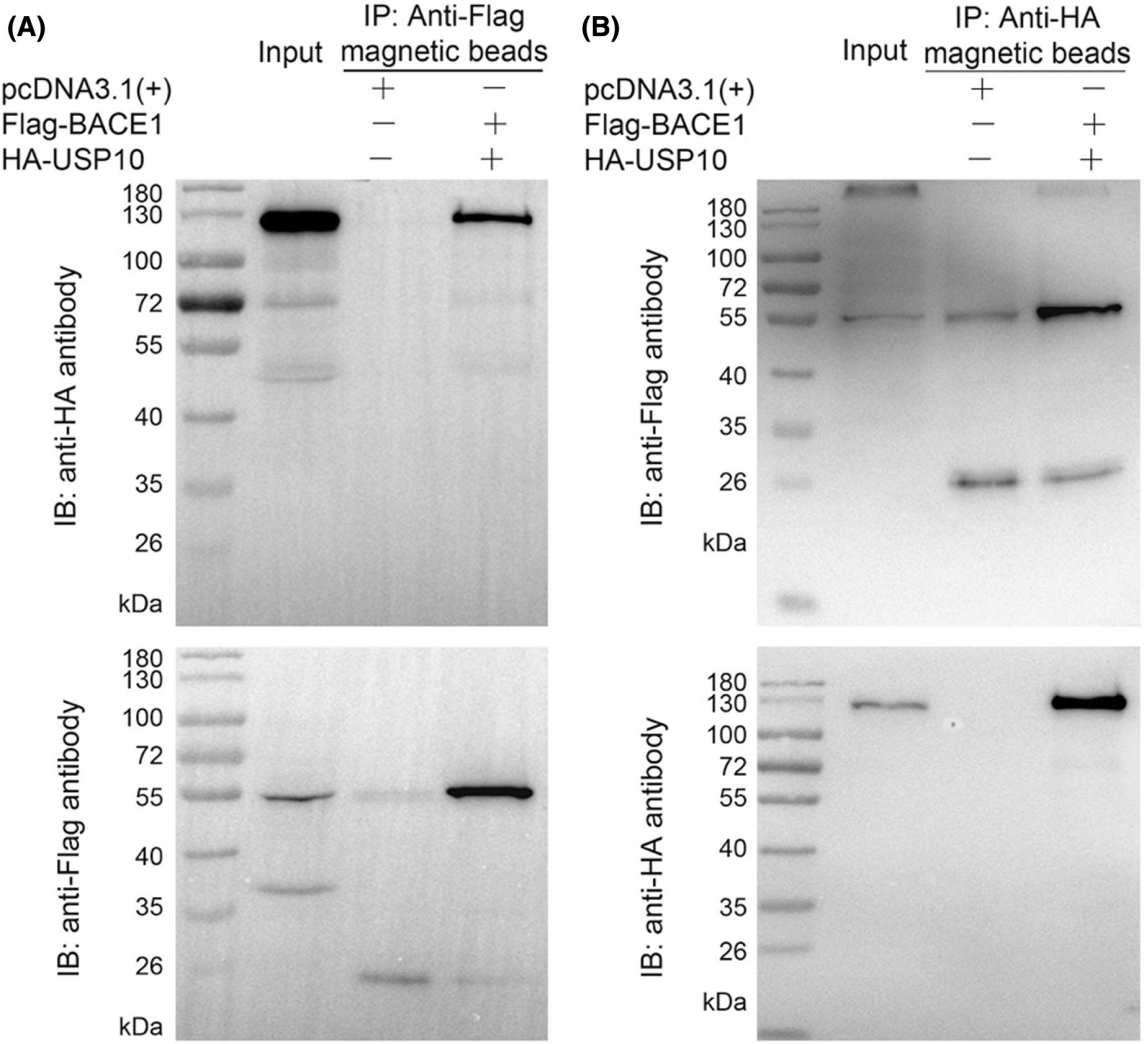
SDS‐PAGE analysis results of BACE1 and USP10 interaction. (A): HA‐USP10 band was detected in the pull‐down complex. (B): Flag‐BACE1 band was detected in the pull‐down complex.

USP10 (NP_001259004.1) was selected for the following Co‐IP experiment based on the results of the docking and MD simulation. The results of the sodium dodecyl sulfate‐polyacrylamide gel electrophoresis (SDS‐PAGE) analysis using hemagglutinin (HA) rabbit polyclonal antibody showed that the HA‐USP10 band was detected in the pull‐down complex (Figure 4A). In addition, the results of western blotting revealed that the Flag‐BACE1 band was detected in the pull‐down complex (Figure 4B). These findings demonstrate that BACE1 interacts with the protein USP10.

### 6.3 The association analysis between the candidated genes on the hippocampal subfields volume and clinical scales

#### 6.3.1 The effects of candiated genes on the hippocampal subfields volume

Taking *USP10* as an example, *USP10* homozygous variants had smaller standardized GC‐ML‐DG volume in the right hippocampus than *USP10* heterozygous variants (*p*‐value <0.05) (Table 3). Both AD and CN of the 88 heterozygous individuals carrying the *USP10* gene were statistically different in the hippocampal subfields of GC‐ML‐DG on both sides (*p*‐value<0.05) (Table 4). The AD and CN of the 229 individuals with homozygous variants carrying the *USP10* gene were also statistically different in the GC‐ML‐DG hippocampal subfield on both sides (*p*‐value<0.05) (Table 5). Therefore, carrying *USP10* might cause changes in GC‐ML‐DG.

**TABLE 3:** Results of the difference test for the effects of USP10 gene polymorphism on the hippocampal subfields' volume.

Variables	GC (*n* = 88)	CC (*n* = 229)	*p*_value_
Left Hippocampal tail	2.939 e‐04 ± 6.020 e‐05	2.868 e‐04 ± 5.510 e‐05	0.334
Left subiculum	2.392 e‐04 (2.026 e‐04, 2.672 e‐04)	2.219 e‐04 (1.877 e‐04, 2.649 e‐04)	0.112
Left_CA1	3.479 e‐04 ± 5.980 e‐05	3.363 e‐04 ± 6.040 e‐05	0.125
Left hippocampal fissure	9.980 e‐05 (9.020 e‐05, 1.138 e‐04)	1.029 e‐04 (9.040 e‐05, 1.139 e‐04)	0.667
Left presubiculum	1.695 e‐04 ± 3.870 e‐05	1.619 e‐04 ± 3.410 e‐05	0.107
Left parasubiculum	3.460 e‐05 ± 9.400 e‐06	3.490 e‐05 ± 9.400 e‐06	0.789
Left molecular layer HP,	3.072 e‐04 (2.645 e‐04, 3.406 e‐04)	2.886 e‐04 (2.464 e‐04, 3.347 e‐04)	0.061
*Left GC‐ML‐DG*	*1.627 e‐04* (*1.412 e‐04, 1.752 e‐04*)	*1.478 e‐04* (*1.276 e‐04, 1.705 e‐04*)	*0.019*
Left CA3	1.122 e‐04 ± 2.180 e‐05	1.077 e‐04 ± 2.190 e‐05	0.099
Left CA4	1.407 e‐04 (1.229 e‐04, 1.538 e‐04)	1.302 e‐04 (1.134 e‐04, 1.491 e‐04)	0.032
Left fimbria	3.110 e‐05 (1.740 e‐05, 3.920 e‐05)	2.740 e‐05 (1.830 e‐05, 3.860 e‐05)	0.382
Left HATA	3.060 e‐05 ± 7.900 e‐06	3.040 e‐05 ± 7.900 e‐06	0.780
Right Hippocampal_tail	3.218 e‐04 ± 5.720 e‐05	3.054 e‐04 ± 5.850 e‐05	0.024
Right subiculum	2.460 e‐04 (2.058 e‐04, 2.795 e‐04)	2.229 e‐04 (1.887 e‐04, 2.625 e‐04)	0.014
Right CA1	3.675 e‐04 ± 6.620 e‐05	3.498 e‐04 ± 6.540 e‐05	0.035
Right hippocampal fissure	1.080 e‐04 (1.010 e‐04, 1.221 e‐04)	1.124 e‐04 (9.710 e‐05, 1.264 e‐04)	0.779
Right presubiculum	1.645 e‐04 (1.358 e‐04, 1.859 e‐04)	1.507 e‐04 (1.286 e‐04, 1.785 e‐04)	0.032
Right parasubiculum	3.370 e‐05 ± 1.000 e‐05	3.350 e‐05 ± 9.300 e‐06	0.891
Right molecular layer HP	3.261 e‐04 (2.793 e‐04, 3.601 e‐04)	2.962 e‐04 (2.548 e‐04, 3.42 e‐04)	0.006
*Right GC‐ML‐DG*	*1.722 e‐04 ± 3.150 e‐05*	*1.620 e‐04 ± 2.970 e‐05*	*0.010*
Right CA3	1.275 e‐04 ± 2.490 e‐05	1.199 e‐04 ± 2.400 e‐05	0.015
Right CA4	1.515 e‐04 ± 2.620 e‐05	1.429 e‐04 ± 2.490 e‐05	0.009
Right fimbria	2.950 e‐05 ± 1.410 e‐05	2.730 e‐05 ± 1.210 e‐05	0.206
Right HATA	3.360 e‐05 (2.900 e‐05, 3.820 e‐05)	3.160 e‐05 (2.580 e‐05, 3.760 e‐05)	0.156

*Note*: GC, individuals with heterozygous carrying the USP10 gene; CC, individuals with homozygous variants carrying the USP10 gene.

**TABLE 4:** The results of the difference test for the effects of USP10 heterozygous variants on hippocampal subfields' volume.

Variables	AD (*n* = 37)	CN (*n* = 51)	*p*_value_
Left Hippocampal tail	2.618 e‐04 ± 5.210 e‐05	3.172 e‐04 ± 5.500 e‐05	< 0.001
Left subiculum	1.996 e‐04 ± 4.770 e‐05	2.598 e‐04 ± 3.420 e‐05	< 0.001
Left CA1	3.143 e‐04 ± 6.200 e‐05	3.723 e‐04 ± 4.470 e‐05	< 0.001
Left hippocampal fissure,	9.630 e‐05 (8.290 e‐05, 1.090 e‐04)	1.028 e‐04 (9.460 e‐05, 1.216 e‐04)	0.016
Left presubiculum	1.435 e‐04 ± 3.220 e‐05	1.884 e‐04 ± 3.150 e‐05	< 0.001
Left parasubiculum	3.120 e‐05 ± 9.500 e‐06	3.710 e‐05 ± 8.600 e‐06	0.004
Left molecular layer HP	2.636 e‐04 ± 5.970 e‐05	3.319 e‐04 ± 3.950 e‐05	< 0.001
*Left GC‐ML‐DG*	*1.406 e‐04 ± 3.340 e‐05*	*1.716 e‐04 ± 2.040 e‐05*	*< 0.001*
Left CA3	1.012 e‐04 ± 2.550 e‐05	1.202 e‐04 ± 1.440 e‐05	< 0.001
Left CA4	1.234 e‐04 ± 2.760 e‐05	1.497 e‐04 ± 1.770 e‐05	< 0.001
Left fimbria	2.150 e‐05 ± 1.300 e‐05	3.670 e‐05 ± 1.340 e‐05	< 0.001
Left HATA	2.730 e‐05 ± 8.600 e‐06	3.310 e‐05 ± 6.300 e‐06	0.001
Right Hippocampal tail	2.880 e‐04 ± 5.670 e‐05	3.463 e‐04 ± 4.390 e‐05	< 0.001
Right subiculum	2.052 e‐04 ± 4.770 e‐05	2.669 e‐04 ± 3.240 e‐05	< 0.001
Right CA1	3.248 e‐04 ± 6.830 e‐05	3.984 e‐04 ± 4.430 e‐05	< 0.001
Right hippocampal fissure	1.058 e‐04 (9.130 e‐05, 1.203 e‐04)	1.140 e‐04 (1.033 e‐04, 1.240 e‐04)	0.078
Right presubiculum	1.383 e‐04 ± 3.190 e‐05	1.792 e‐04 ± 2.700 e‐05	< 0.001
Right parasubiculum	3.020 e‐05 ± 1.050 e‐05	3.630 e‐05 ± 9.000 e‐06	0.005
Right molecular layer HP	2.768 e‐04 ± 6.300 e‐05	3.500 e‐04 ± 3.760 e‐05	< 0.001
*Right GC‐ML‐DG*	*1.541 e‐04 ± 3.490 e‐05*	*1.854 e‐04 ± 2.080 e‐05*	*< 0.001*
Right CA3,	1.144 e‐04 (9.320 e‐05, 1.337 e‐04)	1.348 e‐04 (1.262 e‐04, 1.481 e‐04)	< 0.001
Right CA4	1.369 e‐04 ± 2.940 e‐05	1.621 e‐04 ± 1.730 e‐05	< 0.001
Right fimbria	2.360 e‐05 ± 1.420 e‐05	3.380 e‐05 ± 1.250 e‐05	< 0.001
Right HATA	2.960 e‐05 ± 9.400 e‐06	3.660 e‐05 ± 5.300 e‐06	< 0.001

**TABLE 5:** The results of the difference test for the effects of USP10 homogenous variants on hippocampal subfields' volume.

Variables	AD (*n* = 109)	CN (*n* = 120)	*p*_value_
Left Hippocampal tail	2.544 e‐04 ± 3.590 e‐05	3.162 e‐04 ± 5.310 e‐05	< 0.001
Left subiculum	1.877 e‐04 (1.683 e‐04, 2.086 e‐04)	2.604 e‐04 (2.307 e‐04, 2.784 e‐04)	< 0.001
Left CA1	2.914 e‐04 (2.652 e‐04, 3.181 e‐04)	3.748 e‐04 (3.385 e‐04, 4.100 e‐04)	< 0.001
Left hippocampal fissure	9.850 e‐05 (8.760 e‐05, 1.087 e‐04)	1.059 e‐04 (9.480 e‐05, 1.192 e‐04)	< 0.001
Left presubiculum	1.372 e‐04 (1.260 e‐04, 1.485 e‐04)	1.822 e‐04 (1.665 e‐04, 1.999 e‐04)	< 0.001
Left parasubiculum	3.200 e‐05 ± 9.300 e‐06	3.760 e‐05 ± 8.800 e‐06	< 0.001
left molecular layer HP	2.480 e‐04 (2.256 e‐04, 2.608 e‐04)	3.294 e‐04 (3.004 e‐04, 3.554 e‐04)	< 0.001
*Left GC‐ML‐DG*	*1.299 e‐04* (*1.183 e‐04, 1.427 e‐04*)	*1.678 e‐04* (*1.505 e‐04, 1.803 e‐04*)	*< 0.001*
Left CA3	9.480 e‐05 ± 1.630 e‐05	1.194 e‐04 ± 1.960 e‐05	< 0.001
Left CA4	1.162 e‐04 (1.049 e‐04, 1.271 e‐04)	1.476 e‐04 (1.332 e‐04, 1.572 e‐04)	< 0.001
Left fimbria	2.090 e‐05 (1.310 e‐05, 2.920 e‐05)	3.440 e‐05 (2.510 e‐05, 4.340 e‐05)	< 0.001
Left HATA	2.640 e‐05 ± 7.40 e‐06	3.4.00 e‐05 ± 6.600 e‐06	< 0.001
Right Hippocampal tail	2.647 e‐04 (2.457 e‐04, 2.944 e‐04)	3.308 e‐04 (3.055 e‐04, 3.668 e‐04)	< 0.001
Right subiculum	1.910 e‐04 ± 3.730 e‐05	2.571 e‐04 ± 3.850 e‐05	< 0.001
Right CA1	3.102 e‐04 ± 5.160 e‐05	3.858 e‐04 ± 5.500 e‐05	< 0.001
Right hippocampal fissure	1.077 e‐04 ± 2.320 e‐05	1.166 e‐04 ± 2.350 e‐05	0.004
Right presubiculum	1.330 e‐04 ± 2.570 e‐05	1.711 e‐04 ± 2.870 e‐05	< 0.001
Right parasubiculum	3.100 e‐05 ± 9.200 e‐06	3.580 e‐05 ± 8.900 e‐06	< 0.001
Right molecular layer HP	2.586 e‐04 ± 4.350 e‐05	3.350 e‐04 ± 4.760 e‐05	< 0.001
*Right GC‐ML‐DG*	*1.442 e‐04 ± 2.310 e‐05*	*1.783 e‐04 ± 2.550 e‐05*	*< 0.001*
Right CA3	1.067 e‐04 ± 1.970 e‐05	1.319 e‐04 ± 2.120 e‐05	< 0.001
Right CA4	1.279 e‐04 ± 1.990 e‐05	1.564 e‐04 ± 2.100 e‐05	< 0.001
Right fimbria	2.310 e‐05 ± 1.120 e‐05	3.120 e‐05 ± 1.160 e‐05	< 0.001
Right HATA	2.740 e‐05 (2.300 e‐05, 3.100 e‐05)	3.590 e‐05 (3.100 e‐05, 4.250 e‐05)	< 0.001

The details about the other 4 candidated genes effects on the hippocampal subfields volume are described in the Supplementary Materials Tables S4–S21. For instance, carrying *MRGPRX3* might cause changes in right GC‐ML‐DG(Tables S4–S9, *p*‐value<0.05). Carrying *NDUFA11* might cause changes in left GC‐ML‐DG and left CA3(Tables S10–S12, *p*‐value<0.05).

#### 6.3.2 The association between the candiated genes and cliniccal scales

Table 6 described the significant differences between homozygous variants and heterozygous variants of the *USP10* gene on the MMSE score and GDS score (*p*‐value <0.05). Therefore, AD patient with depressive symptoms was associated with homozygous variants carrying the *USP10* gene.

**TABLE 6:** Results of the association analysis between the candiated genes and cliniccal scales.

gene	Scale name	Items	group	Heterozygous variant	Homozygous variant	*p*_value_
*USP10*	MMSE	Spell “world” backward (letter O)	Incorrect	67	200	0.023
			Correct	21	29	
	GDS	Do you think that most people are better off than you are?	Incorrect	82	225	0.031
			Correct	6	4	
*TRPV1*	MMSE	The subject was asked to repeat the word “Flag”	Incorrect	67	97	0.026
			Correct	74	62	
		The subject was asked to repeat the word “Tree”	Incorrect	70	100	0.028
			Correct	71	59	
		Now I am going to ask you to repeat what I say	Incorrect	114	143	0.038
			Correct	27	16	
	GDS	Do you have any trouble with memory?	Incorrect	91	126	0.007
			Correct	50	33	
		Do you think it is wonderful to be alive right now	Incorrect	9	1	0.007
			Correct	132	158	
*MRGPRX3*	MMSE	Now I am going to ask you to repeat what I say	Incorrect	39	245	0.011
			Correct	13	30	
		The subjects were asked to copy two intersecting pentagon shapes drawn on the white paper	Incorrect	35	222	0.048
			Correct	17	53	
			Correct	17	53	
*SEPT9*	MMSE	Spell “world” backward (letter W)	Incorrect	143	116	0.036
			Correct	4	12	
*NDUFA11*	MMSE	Please read this and do what it says	Incorrect	39	280	0.007
			Correct	3	1	

The details about the association between other 4 candidated genes and clinical scales are displayed in the Table 6. For patients with AD, delay recall and depressive symptoms were associated with *TRPV1* gene; precise repetition was linked with *MRGPRX3* gene; an aggravation of spelling errors was related with *SEPT9* gene; and reading disorder was associated with *NDUFA11* gene.

## 7 DISCUSSION

Degeneration of adrenergic neurons in locus coeruleus of brainstem and/or of serotonergic neurons sends projections to cerebral cortex and hippocampus and leads to impaired metabolic and functional interactions of neurons in the hippocampus.^3^, ^67^, ^68^ According to four distinct spatiotemporal trajectories of tau pathology, one of the four subtypes of AD accounted for the most (33%) is subtype of tau that spreads within the temporal lobe and affects memory.^69^ The hippocampus in the temporal lobe compries histologically and unique functional distinguishable subfields with differential vulnerability to AD. The hippocampus is subdivided by using FreeSufer due to the cytoarchitecture of the hippocampal subfields. Using brain scans in the ADNI dataset, we demonstrated that the difference of hippocampal subfields was affected by difference in their genetic architecture. The identification on genetic architecture and specific genetic variants on hippocampal subfields is useful to better understand the underlying biological functions of subfields and their roles in the development of AD.

We identified several genetic variants (*USP10*, *TRPV1*, *NDUFA11*, *MRGPRX3*, and *SEPT9*) associated with the volumes of the hippocampal subfields. This findings also largely agree with previous studies. For example, the upregulation of *TRPV1* leads to neuronal death in the hippocampus and is involved in the modulation of synaptic plasticity.^70^, ^71^, ^72^ The atrophy of synapses between the cortex and hippocampus has been shown to be caused by the reduction in CA4 volume.^73^ For *NDUFA11*, mutations in *NDUFA11* are associated with severe mitochondrial complex I deficiency. Mitochondrial complex I dysfunction accelerates amyloid toxicity and mitochondrial complex I dysfunction in aging, which may contribute to the pathogenesis of sporadic AD.^74^ SEPT9 interacts with kinesin KIF17 and interferes with the mechanism of NMDA receptor cargo binding and transport. Hippocampal NMDA receptors might be involved in neurobehavioral abnormalities via inflammation in sporadic AD.^75^ Aβ influences N‐methyl d‐aspartate (NMDA) receptor activation in AD, which is presented in hippocampal DG granule cells, CA3, CA4.^76^, ^77^, ^78^, ^79^

The results of homology modeling, molecular docking, MD simulations, and Co‐IP experiments show that USP10 has the strongest interaction with BACE1 among five identified genes.*USP10* is a member of the USP domain family of deubiquitinating enzymes (DUB, a new therapeutic target in cases of neurodegenerative diseases^80^), which comprises over 50 members, including *USP8* and *USP25*.^81^, ^82^ The study by Yeates et al. demonstrated that BACE1 was a direct substrate of USP8 deubiquitination and induces an increase in Aβ.^83^ Zheng's study demonstrated that USP25 promoted the cleavage of APP as well as the generation of Aβ through deubiquitination of BACE1.^82^ Our finding is also consistent with previous reports of reduced USP10 activity, decreasing Aβ secretion to ameliorate Aβ plaque load and improving deficits in learning memory.^84^

The results of association analysis between the candidated genes on the hippocampal subfields volume and clinical scales showed that candidated genes influenced the volume and function of hippocampal subfields. Taking *USP10* as an example, homozygous variants of *USP10* had smaller standardized granule cell and molecular layer of the dentate gyrus (GC‐ML‐DG) volume in the right hippocampus than *USP10* heterozygous variants (*p*‐value <0.05). And homozygous variants were statistically different compared to heterozygous variants on the cognitive scale (*p*‐value <0.05). GC‐ML‐DG volume was found to be smaller in patients with MCI or early MCI compared with CN.^85^ The cause of GC‐ML‐DG atrophy is associated with abnormal Aβ1–42 and P‐Tau181 (A + T+) in AD patients and MCI subjects.^85^ In the AD group, G. Šimić et.al found a significant loss of neurons in the DG (https://pubmed.ncbi.nlm.nih.gov/9067838/).

However, our study has several limitations. Firstly, the use of different MRI scanner types from different centers may result in bias. Secondly, our small sample size limits the generalizability of our results. In addition, we only investigated the interaction between USP10 and BACE1. Additional genes for AD will likely be identified if other proteins related to AD besides BACE1 are included.

Taken together, the involvement of *USP10* in the pathological and molecular mechanisms underlying AD is preliminarily demonstrated by the MD and CO‐IP experiment, and warrants further exploration.

In conclusion, we identify novel non‐synonymous variants that influenced specific hippocampal subfields and demonstrate that difference genetic architecture on hippocampal subfields, associated with specific biological processes and functions, showing that a greater specificity of the hippocampal subfields is existed. We believe that the specificity may help us to understand the underlying hippocampal neurobiology and its related functions in AD.

## AUTHOR CONTRIBUTIONS

HT conceived and oversaw the project, JC and XW contributed to the data analysis, and MD simulation and performed the bioinformatics analysis. WX carried out the Co‐IP experiments. All authors made critical contributions to the manuscript.

## FUNDING INFORMATION

Our research was supported by the Third Medical Technology Projects of Shantou in 2018(41368043); the Natural Science Foundation of China (11771462, 71991474, 72171216), the National Key Research, the Key Research and Development Program of Guangdong, China (2019B020228001), the Science and Technology Program of Guangzhou, China (Grant No. 202002030129), and the Natural Science Foundation of Anhui (BJ2040170017).

## CONFLICT OF INTEREST STATEMENT

The authors declare no conflicts of interest.

## CODE AVAILABILITY STATEMENTS

All code used for data cleaning and analysis is publically accessible at: https://github.com/stu5.

## CONSENT TO PARTICIPATE

All consent documentation is available on the ADNI website (http://adni.loni.usc.edu/methods/documents/).

## Supporting information


Tables S1‐S21

## Data Availability

Data used in this research were obtained from the ADNI database (http://adni.loni.usc.edu), which is an open‐source database.

## References

[1] Anblagan D , Valdés Hernández MC , Ritchie SJ , et al. Coupled changes in hippocampal structure and cognitive ability in later life. Brain Behav. 2018;8(2):e00838. 29484252 10.1002/brb3.838 PMC5822578

[2] Van der Meer D , Rokicki J , Kaufmann T , et al. Brain scans from 21,297 individuals reveal the genetic architecture of hippocampal subfield volumes. Mol Psychiatry. 2020;25(11):3053‐3065. 30279459 10.1038/s41380-018-0262-7 PMC6445783

[3] Hertz L . Is Alzheimer's disease an anterograde degeneration, originating in the brainstem, and disrupting metabolic and functional interactions between neurons and glial cells? Brain Res Rev. 1989;14(4):335‐353. 2696574 10.1016/0165-0173(89)90017-9

[4] Guo T , Landau SM , Jagust WJ . Age, vascular disease, and Alzheimer's disease pathologies in amyloid negative elderly adults. Alzheimers Res Ther. 2021;13(1):1‐12. 34654465 10.1186/s13195-021-00913-5 PMC8520216

[5] Wu J , Dong Q , Zhang J , et al. Federated morphometry feature selection for hippocampal morphometry associated beta‐amyloid and tau pathology. Front Neurosci. 2021;15:762458. 34899166 10.3389/fnins.2021.762458 PMC8655732

[6] Hanko V , Apple AC , Alpert KI , et al. In vivo hippocampal subfield shape related to TDP‐43, amyloid beta, and tau pathologies. Neurobiol Aging. 2019;74:171‐181. 30453234 10.1016/j.neurobiolaging.2018.10.013 PMC6331233

[7] Mattsson N , Insel PS , Aisen PS , et al. Brain structure and function as mediators of the effects of amyloid on memory. Neurology. 2015;84(11):1136‐1144. 25681451 10.1212/WNL.0000000000001375 PMC4371407

[8] Roda AR , Montoliu‐Gaya L , Serra‐Mir G , Villegas S . Both amyloid‐β peptide and tau protein are affected by an anti‐amyloid‐β antibody fragment in elderly 3xTg‐AD mice. Int J Mol Sci. 2020;21(18):6630. 32927795 10.3390/ijms21186630 PMC7554787

[9] Duarte A , Hayasaka S , Du A , et al. Volumetric correlates of memory and executive function in normal elderly, mild cognitive impairment and Alzheimer's disease. Neurosci Lett. 2006;406(1–2):60‐65. 16904823 10.1016/j.neulet.2006.07.029 PMC1779764

[10] Bartel F , Visser M , de Ruiter M , et al. Non‐linear registration improves statistical power to detect hippocampal atrophy in aging and dementia. Neuroimage Clin. 2019;23:101902. 31233953 10.1016/j.nicl.2019.101902 PMC6595082

[11] DeTure MA , Dickson DW . The neuropathological diagnosis of Alzheimer's disease. Mol Neurodegener. 2019;14(1):1‐18. 31375134 10.1186/s13024-019-0333-5 PMC6679484

[12] Zhao W , Wang X , Yin C , He M , Li S , Han Y . Trajectories of the hippocampal subfields atrophy in the Alzheimer's disease: a structural imaging study. Front Neuroinform. 2019;13:13. 30983985 10.3389/fninf.2019.00013 PMC6450438

[13] Cipriani S , Journiac N , Nardelli J , et al. Dynamic expression patterns of progenitor and neuron layer markers in the developing human dentate gyrus and fimbria. Cereb Cortex. 2017;27(1):358‐372. 26443441 10.1093/cercor/bhv223 PMC5894254

[14] La Joie R , Perrotin A , de La Sayette V , et al. Hippocampal subfield volumetry in mild cognitive impairment, Alzheimer's disease and semantic dementia. Neuroimage Clin. 2013;3:155‐162. 24179859 10.1016/j.nicl.2013.08.007 PMC3791274

[15] Izzo J , Andreassen OA , Westlye LT , van der Meer D . The association between hippocampal subfield volumes in mild cognitive impairment and conversion to Alzheimer's disease. Brain Res. 2020;1728:146591. 31816319 10.1016/j.brainres.2019.146591

[16] Fukutani Y , Cairns NJ , Shiozawa M , et al. Neuronal loss and neurofibrillary degeneration in the hippocampal cortex in late‐onset sporadic Alzheimer's disease. Psychiatry Clin Neurosci. 2000;54(5):523‐529. 11043800 10.1046/j.1440-1819.2000.00747.x

[17] West MJ , Coleman PD , Flood DG , Troncoso JC . Differences in the pattern of hippocampal neuronal loss in normal ageing and Alzheimer's disease. The Lancet. 1994;344(8925):769‐772. 10.1016/s0140-6736(94)92338-8 7916070

[18] Adler DH , Wisse LE , Ittyerah R , et al. Characterizing the human hippocampus in aging and Alzheimer's disease using a computational atlas derived from ex vivo MRI and histology. Proc Natl Acad Sci. 2018;115(16):4252‐4257. 29592955 10.1073/pnas.1801093115 PMC5910869

[19] Tesli N , van der Meer D , Rokicki J , et al. Hippocampal subfield and amygdala nuclei volumes in schizophrenia patients with a history of violence. Eur Arch Psychiatry Clin Neurosci. 2020;270(6):771‐782. 31980898 10.1007/s00406-020-01098-y PMC7423802

[20] Shi Y , Cheng K , Liu Z . Hippocampal subfields segmentation in brain MR images using generative adversarial networks. Biomed Eng Online. 2019;18(1):1‐12. 30665408 10.1186/s12938-019-0623-8 PMC6341719

[21] Tao S , Wang Y , Peng J , et al. Whole‐brain mapping the direct inputs of dorsal and ventral CA1 projection neurons. Front Neural Circuits. 2021;15:643230. 33935658 10.3389/fncir.2021.643230 PMC8079783

[22] Tanaka KZ . Heterogeneous representations in the hippocampus. Neurosci Res. 2021;165:1‐5. 32445753 10.1016/j.neures.2020.05.002

[23] Armstrong NM , Dumitrescu L , Huang C‐W , et al. Association of hippocampal volume polygenic predictor score with baseline and change in brain volumes and cognition among cognitively healthy older adults. Neurobiol Aging. 2020;94:81‐88. 32593031 10.1016/j.neurobiolaging.2020.05.007 PMC8893954

[24] De Flores R , La Joie R , Chételat G . Structural imaging of hippocampal subfields in healthy aging and Alzheimer's disease. Neuroscience. 2015;309:29‐50. 26306871 10.1016/j.neuroscience.2015.08.033

[25] Wang SY , Xue X , Duan R , et al. A TREML2 missense variant influences specific hippocampal subfield volumes in cognitively normal elderly subjects. Brain Behav. 2020;10(4):e01573. 32073739 10.1002/brb3.1573 PMC7177563

[26] Elman JA , Panizzon MS , Gillespie NA , et al. Genetic architecture of hippocampal subfields on standard resolution MRI: how the parts relate to the whole. Hum Brain Mapp. 2019;40(5):1528‐1540. 30430703 10.1002/hbm.24464 PMC6397064

[27] Ambrée O , Buschert J , Zhang W , Arolt V , Dere E , Zlomuzica A . Impaired spatial learning and reduced adult hippocampal neurogenesis in histamine H1‐receptor knockout mice. Eur Neuropsychopharmacol. 2014;24(8):1394‐1404. 24862254 10.1016/j.euroneuro.2014.04.006

[28] Keskin O , Tuncbag N , Gursoy A . Predicting protein–protein interactions from the molecular to the proteome level. Chem Rev. 2016;116(8):4884‐4909. 27074302 10.1021/acs.chemrev.5b00683

[29] Chong WL , Chupradit K , Chin SP , et al. Protein‐protein interactions: insight from molecular dynamics simulations and nanoparticle tracking analysis. Molecules. 2021;26(18):5696. 34577167 10.3390/molecules26185696 PMC8472368

[30] Wang Z , Huang X , Zhao P , Zhao L , Wang Z‐Y . Catalpol inhibits amyloid‐β generation through promoting α‐cleavage of APP in Swedish mutant APP overexpressed N2a cells. Front Aging Neurosci. 2018;10:66. 29615891 10.3389/fnagi.2018.00066 PMC5867310

[31] Zhao L , Zhao Y , Tang F‐L , et al. pHluorin‐BACE1‐mCherry acts as a reporter for the intracellular distribution of active BACE1 in vitro and in vivo. Cell. 2019;8(5):474. 10.3390/cells8050474 PMC6562731 31108937

[32] Marwarha G , Schommer J , Lund J , Schommer T , Ghribi O . Palmitate‐induced C/EBP homologous protein activation leads to NF‐κB‐mediated increase in BACE1 activity and amyloid beta genesis. J Neurochem. 2018;144(6):761‐779. 29315574 10.1111/jnc.14292 PMC6371812

[33] Yi‐Bin W , Xiang L , Bing Y , et al. Inhibition of the CEBPβ‐NFκB interaction by nanocarrier‐packaged Carnosic acid ameliorates glia‐mediated neuroinflammation and improves cognitive function in an Alzheimer's disease model. Cell Death Dis. 2022;13(4):1‐18. 10.1038/s41419-022-04765-1 PMC8989877 35393391

[34] Lomoio S , Willen R , Kim W , et al. Gga3 deletion and a GGA3 rare variant associated with late onset Alzheimer's disease trigger BACE1 accumulation in axonal swellings. Sci Transl Med. 2020;12(570):eaba1871. 33208500 10.1126/scitranslmed.aba1871 PMC8612295

[35] Buggia‐Prévot V , Fernandez CG , Riordan S , et al. Axonal BACE1 dynamics and targeting in hippocampal neurons: a role for Rab11 GTPase. Mol Neurodegener. 2014;9(1):1‐18. 24386896 10.1186/1750-1326-9-1 PMC4031619

[36] Kandalepas PC , Sadleir KR , Eimer WA , Zhao J , Nicholson DA , Vassar R . The Alzheimer's β‐secretase BACE1 localizes to normal presynaptic terminals and to dystrophic presynaptic terminals surrounding amyloid plaques. Acta Neuropathol. 2013;126(3):329‐352. 23820808 10.1007/s00401-013-1152-3 PMC3753469

[37] Fujihara K , Takei Y . Freesurfer as a platform for associating brain structure with function. Brain Nerve. 2018;70(7):841‐848. 29997280 10.11477/mf.1416201085

[38] Kumar P , Henikoff S , Ng PC . Predicting the effects of coding non‐synonymous variants on protein function using the SIFT algorithm. Nat Protoc. 2009;4(7):1073‐1081. 19561590 10.1038/nprot.2009.86

[39] Pipitone J , Park MTM , Winterburn J , et al. Multi‐atlas segmentation of the whole hippocampus and subfields using multiple automatically generated templates. Neuroimage. 2014;101:494‐512. 24784800 10.1016/j.neuroimage.2014.04.054

[40] Iglesias JE , Augustinack JC , Nguyen K , et al. A computational atlas of the hippocampal formation using ex vivo, ultra‐high resolution MRI: application to adaptive segmentation of in vivo MRI. Neuroimage. 2015;115:117‐137. 25936807 10.1016/j.neuroimage.2015.04.042 PMC4461537

[41] HippocampalSubfieldsAndNucleiOfAmygdala. https://surfer.nmr.mgh.harvard.edu/fswiki/

[42] Sämann PG , Iglesias JE , Gutman B , et al. FreeSurfer‐based segmentation of hippocampal subfields: a review of methods and applications, with a novel quality control procedure for ENIGMA studies and other collaborative efforts. Hum Brain Mapp. 2022;43(1):207‐233. 33368865 10.1002/hbm.25326 PMC8805696

[43] Li H , Zhao L , Zhang M . Gut microbial SNPs induced by high‐fiber diet dominate nutrition metabolism and environmental adaption of Faecalibacterium prausnitzii in obese children. Front Microbiol. 2021;12:683714. 34135881 10.3389/fmicb.2021.683714 PMC8200495

[44] Laurie CC , Doheny KF , Mirel DB , et al. Quality control and quality assurance in genotypic data for genome‐wide association studies. Genet Epidemiol. 2010;34(6):591‐602. 20718045 10.1002/gepi.20516 PMC3061487

[45] Carlton VE , Ireland JS , Useche F , Faham M . Functional single nucleotide polymorphism‐based association studies. Hum Genomics. 2006;2(6):1‐12. 10.1186/1479-7364-2-6-391 PMC3525158 16848977

[46] Price AL , Patterson NJ , Plenge RM , Weinblatt ME , Shadick NA , Reich D . Principal components analysis corrects for stratification in genome‐wide association studies. Nat Genet. 2006;38(8):904‐909. 16862161 10.1038/ng1847

[47] Nazarian A , Yashin AI , Kulminski AM . Genome‐wide analysis of genetic predisposition to Alzheimer's disease and related sex disparities. Alzheimers Res Ther. 2019;11(1):5. 30636644 10.1186/s13195-018-0458-8 PMC6330399

[48] Sun Y , Guo Y , Feng X , et al. The behavioural and neuropathologic sexual dimorphism and absence of MIP‐3α in tau P301S mouse model of Alzheimer's disease. J Neuroinflammation. 2020;17(1):1‐18. 32093751 10.1186/s12974-020-01749-w PMC7041244

[49] Tesseur I , Lo A , Roberfroid A , et al. Bexarotene treatment does not clear β‐amyloid in an AD mouse model and beagle dogs. Mol Neurodegener. 2013;8(1):1.23281774

[50] Van Bergen J , Li X , Hua J , et al. Colocalization of cerebral iron with amyloid beta in mild cognitive impairment. Sci Rep. 2016;6(1):1‐9. 27748454 10.1038/srep35514 PMC5066274

[51] Wen C , Ba H , Pan W , Huang M , AsDN I . Co‐sparse reduced‐rank regression for association analysis between imaging phenotypes and genetic variants. Bioinformatics. 2020;36(21):5214‐5222. 10.1093/bioinformatics/btaa650 PMC7849977 32683450

[52] Miyamoto M , Kuzuya A , Noda Y , et al. Synaptic vesicle protein 2B negatively regulates the Amyloidogenic processing of AβPP as a novel interaction partner of BACE1. J Alzheimers Dis. 2020;75(1):173‐185. 32280101 10.3233/JAD-200071

[53] Zhang Y‐w , Thompson R , Zhang H , Xu H . APP processing in Alzheimer's disease. Mol Brain. 2011;4(1):1‐13. 21214928 10.1186/1756-6606-4-3 PMC3022812

[54] Okada H , Zhang W , Peterhoff C , et al. Proteomic identification of sorting nexin 6 as a negative regulator of BACE1‐mediated APP processing. FASEB J. 2010;24(8):2783‐2794. 20354142 10.1096/fj.09-146357 PMC2909280

[55] Wolfe MS . γ‐Secretase as a target for Alzheimer's disease. Adv Pharmacol. 2012;64:127‐153. 22840746 10.1016/B978-0-12-394816-8.00004-0

[56] Maramai S , Benchekroun M , Gabr MT , Yahiaoui S . Multitarget therapeutic strategies for Alzheimer's disease: review on emerging target combinations. Biomed Res Int. 2020;2020:1‐27. 10.1155/2020/5120230 PMC7354643 32714977

[57] Ghosh AK , Osswald HL . BACE1 (β‐secretase) inhibitors for the treatment of Alzheimer's disease. Chem Soc Rev. 2014;43(19):6765‐6813. 24691405 10.1039/c3cs60460h PMC4159447

[58] Vagnoni A , Perkinton MS , Gray EH , Francis PT , Noble W , Miller CC . Calsyntenin‐1 mediates axonal transport of the amyloid precursor protein and regulates Aβ production. Hum Mol Genet. 2012;21(13):2845‐2854. 22434822 10.1093/hmg/dds109 PMC3373235

[59] Buchete N‐V , Hummer G . Structure and dynamics of parallel β‐sheets, hydrophobic core, and loops in Alzheimer's Aβ fibrils. Biophys J. 2007;92(9):3032‐3039. 17293399 10.1529/biophysj.106.100404 PMC1852365

[60] Armstrong NM , Huang C‐W , Williams OA , et al. Sex differences in the association between amyloid and longitudinal brain volume change in cognitively normal older adults. Neuroimage Clin. 2019;22:101769. 30927602 10.1016/j.nicl.2019.101769 PMC6444285

[61] Yang J , Yan R , Roy A , Xu D , Poisson J , Zhang Y . The I‐TASSER suite: protein structure and function prediction. Nat Methods. 2015;12(1):7‐8. 10.1038/nmeth.3213 PMC4428668 25549265

[62] Pettersen EF , Goddard TD , Huang CC , et al. UCSF chimera—a visualization system for exploratory research and analysis. J Comput Chem. 2004;25(13):1605‐1612. 15264254 10.1002/jcc.20084

[63] Wiederstein M , Sippl MJ . ProSA‐web: interactive web service for the recognition of errors in three‐dimensional structures of proteins. Nucleic Acids Res. 2007;35(suppl_2):W407‐W410. 17517781 10.1093/nar/gkm290 PMC1933241

[64] Dominguez C , Boelens R , Bonvin AM . HADDOCK: a protein− protein docking approach based on biochemical or biophysical information. J Am Chem Soc. 2003;125(7):1731‐1737. 12580598 10.1021/ja026939x

[65] Hassan M , Shahzadi S , Seo SY , Alashwal H , Zaki N , Moustafa AA . Molecular docking and dynamic simulation of AZD3293 and solanezumab effects against BACE1 to treat Alzheimer's disease. Front Comput Neurosci. 2018;12:34. 29910719 10.3389/fncom.2018.00034 PMC5992503

[66] Ali S , Khan FI , Mohammad T , Lan D , Hassan M , Wang Y . Identification and evaluation of inhibitors of lipase from Malassezia restricta using virtual high‐throughput screening and molecular dynamics studies. Int J Mol Sci. 2019;20(4):884. 30781686 10.3390/ijms20040884 PMC6412828

[67] key facts of dementia . Accessed 2 September, 2021. Available online: https://www.who.int/news‐room/fact‐sheets/detail/dementia

[68] Muresan Z , Muresan V . Seeding neuritic plaques from the distance: a possible role for brainstem neurons in the development of Alzheimer's disease pathology. Neurodegener Dis. 2008;5(3–4):250‐253. 18322404 10.1159/000113716 PMC2562573

[69] Vogel JW , Young AL , Oxtoby NP , et al. Four distinct trajectories of tau deposition identified in Alzheimer's disease. Nat Med. 2021;27(5):871‐881. 33927414 10.1038/s41591-021-01309-6 PMC8686688

[70] Sun F‐J , Guo W , Zheng D‐H , et al. Increased expression of TRPV1 in the cortex and hippocampus from patients with mesial temporal lobe epilepsy. J Mol Neurosci. 2013;49(1):182‐193. 22936245 10.1007/s12031-012-9878-2

[71] Van Spronsen M , Hoogenraad CC . Synapse pathology in psychiatric and neurologic disease. Curr Neurol Neurosci Rep. 2010;10(3):207‐214. 20425036 10.1007/s11910-010-0104-8 PMC2857788

[72] Kim J , Lee S , Kim J , et al. Ca2+−permeable TRPV1 pain receptor knockout rescues memory deficits and reduces amyloid‐β and tau in a mouse model of Alzheimer's disease. Hum Mol Genet. 2020;29(2):228‐237. 31814000 10.1093/hmg/ddz276

[73] Cao B , Passos IC , Mwangi B , et al. Hippocampal subfield volumes in mood disorders. Mol Psychiatry. 2017;22(9):1352‐1358. 28115740 10.1038/mp.2016.262 PMC5524625

[74] Joh Y , Choi W‐S . Mitochondrial complex I inhibition accelerates amyloid toxicity. Dev Reprod. 2017;21(4):417‐424. 29354787 10.12717/DR.2017.21.4.417 PMC5769135

[75] Amani M , Zolghadrnasab M , Salari A‐A . NMDA receptor in the hippocampus alters neurobehavioral phenotypes through inflammatory cytokines in rats with sporadic Alzheimer‐like disease. Physiol Behav. 2019;202:52‐61. 30641081 10.1016/j.physbeh.2019.01.005

[76] Karthick C , Nithiyanandan S , Essa MM , Guillemin GJ , Jayachandran SK , Anusuyadevi M . Time‐dependent effect of oligomeric amyloid‐β (1–42)‐induced hippocampal neurodegeneration in rat model of Alzheimer's disease. Neurol Res. 2019;41(2):139‐150. 30453864 10.1080/01616412.2018.1544745

[77] McHugh TJ , Jones MW , Quinn JJ , et al. Dentate gyrus NMDA receptors mediate rapid pattern separation in the hippocampal network. Science. 2007;317(5834):94‐99. 17556551 10.1126/science.1140263

[78] Christian KM , Miracle AD , Wellman CL , Nakazawa K . Chronic stress‐induced hippocampal dendritic retraction requires CA3 NMDA receptors. Neuroscience. 2011;174:26‐36. 21108993 10.1016/j.neuroscience.2010.11.033 PMC3020251

[79] Zhang X‐y , Liu A‐P , Ruan D‐Y , Liu J . Effect of developmental lead exposure on the expression of specific NMDA receptor subunit mRNAs in the hippocampus of neonatal rats by digoxigenin‐labeled in situ hybridization histochemistry. Neurotoxicol Teratol. 2002;24(2):149‐160. 11943503 10.1016/s0892-0362(01)00210-0

[80] Lim K‐H , Joo J‐Y , Baek K‐H . The potential roles of deubiquitinating enzymes in brain diseases. Ageing Res Rev. 2020;61:101088. 32470641 10.1016/j.arr.2020.101088

[81] Bhattacharya U , Neizer‐Ashun F , Mukherjee P , Bhattacharya R . When the chains do not break: the role of USP10 in physiology and pathology. Cell Death Dis. 2020;11(12):1‐10. 33277473 10.1038/s41419-020-03246-7 PMC7718870

[82] Zheng Q , Song B , Li G , et al. USP25 inhibition ameliorates Alzheimer's pathology through the regulation of APP processing and Aβ generation. J Clin Invest. 2022;132(5):e152170. 35229730 10.1172/JCI152170 PMC8884900

[83] Yeates EFA , Tesco G . The endosome‐associated deubiquitinating enzyme USP8 regulates BACE1 enzyme ubiquitination and degradation. J Biol Chem. 2016;291(30):15753‐15766. 27302062 10.1074/jbc.M116.718023 PMC4957057

[84] Zhang Y , Chen X , Zhao Y , Ponnusamy M , Liu Y . The role of ubiquitin proteasomal system and autophagy‐lysosome pathway in Alzheimer's disease. Rev Neurosci. 2017;28(8):861‐868. 28704199 10.1515/revneuro-2017-0013

[85] Baek MS , Lee N , Xu X , Kim JW , et al. Association of hippocampal subfield volumes with amyloid‐beta deposition in Alzheimer’s disease. Journal of Clinical Medicine. 2022;11(6):1526. 35329851 10.3390/jcm11061526 PMC8955328

