# Supplementary material for: Genetic architecture of hippocampus subfields volumes in Alzheimer’s disease
Source: CNS Neurosci Ther. 2023 Feb 8;30(3):e14110. doi: 10.1111/cns.14110 (PMC10915996; doi:10.1111/cns.14110)
Supplement: Supplementary file 1 — Tables S1‐S21 [file CNS-30-e14110-s001.docx]

Supplementary Material and Methods

**Contents**

1. Gentetic Pre-processing

1. Co-Immunoprecipitation (Co-IP) experiment

3.1 Materials and Reagents

3.2 Transfection

3.3 Co-Immunoprecipitation

3.4 Western blot

3.5 Results

1. The difference analysis for demographics and clinical variables between AD and HC cohorts
2. The association analysis between the candidated genes on the hippocampal subfields volume and clinical scales

**1. Genetic Pre-processing**

There are several procedures before doing imaging-genetic analysis:

(1) Pre-imputation

1,1 Quality Control

We eliminated SNPs with genotype call rate>5%, MAF<5%, samples with call rate > 5% and Hardy-Weinberg equilibrium P < 1.00e-05.

1.2 Shapeit and Phasing.

Converting genome coordinate into hg19 using the UCSC liftOverPlink utility, and phasing were performed using SHAPEIT v2.r904.

（2）Imputation.

Genotype imputation on the Michigan Imputation Sever with Haplotype Reference Consortrium as reference panel (https://imputationserver.sph.umich.edu/index.html#!)

（3）Post-Imputation

3.1 Filter

BCFTOOLS was applied to filter SNPs with R^2^ > 0.8.

3.2 Quality Control

We eliminated SNPs with genotype call rate>5%, MAF<5%, samples with call rate > 5% and Hardy-Weinberg equilibrium P < 1.00e-05.

3.3 Gene-based Annovation

Gene-based annovation was carried out by ANNOVAR (http://www.openbioinformatics.org/annovar/). About 11596 non-synonymous mutations variants are retained.

**2. Co-Immunoprecipitation (Co-IP) experiment**

（1）Materials and Reagents

（2）Transfection

HEK 293T cells in logarithmic growth phase were seeded in 10 cm petri dishes and incubated overnight. Next day, the cells were cotransfected with pCDAN3.1(+)-Flag-BACE1 and pCDAN3.1(+)-HA-USP10 plasmids while pCDAN3.1(+) vector as control. 48 hours after transfection, HEK 293T cells were subjected to co-immunoprecipitation assay.

（3）Co-Immunoprecipitation

Step1: HEK 293T cells gently washed twice in pre-chilled PBS, 5 minutes each wash.

Step2: Added cold NP-40 lysis buffer (1 mL for 10^7^ cells), incubated for 30 minutes on ice.

Step3: Scraped cells off to clean 1.5ml eppendorf tubes with a clean, cold scraper.

Step4: Centrifuged at 14,000 g 4°C for 15 minutes, transfered the supernatant to new tubes.

Step5: Diluted the total protein which is quantified with BCA assay to 2 μg/μ in NP-40 lysis buffer.

Step6: Pipetted 50 μL anti-Flag (or anti-HA) magnetic beads and washed magnetic beads 3 times with cold PBS, softly shaking 5 minuties.

Step7: Mixed 500 μL total protein (1000 μg) with the pre-treated anti-Flag (or anti-HA) magnetic beads (50 μL/tube) and shaked at 4°C overnight.

Step8: Washed the magnetic beads complex with 800 µL NP-40 lysis buffer for three times, 5 minutes each time.

Step9: Added 80 µL 1×SDS loading buffer into each tube and boiled 5 minutes.

（4）Western blot

The protein samples were equally loaded on 10% SDS-PAGE and subsequently transferred to PVDF membrane. After blocking with 5% non-fat milk for 1 hour at room temperature, the whole membranes were incubated with Flag mouse monoclonal antibody and HA rabbit polyclonal antibody respectively. After washing away any outbound primary antibody, the membranes were incubated Horseradish peroxidase labeled secondary antibody again. The signals were detected by ECL detect reagents with Bio-Rad imaging system according to manufacturer’s instructions.

（5）Results of Co-IP experiment results of BACE1 interact with USP10

To verify the interaction between BACE1 and USP10, we co-transfected pCDAN3.1(+)-Flag-BACE1 and pCDAN3.1(+)-HA-USP10 plasmids into HEK 293T cells. After transfection, the HEK 293T cells were subjected to co-immunoprecipitation assays with anti-Flag magnetic beads. SDS-PAGE analysis using HA rabbit polyclonal antibody were subsequently conducted. Results showed that HA-USP10 band was detected in the pull-down complex. In addition, co-immunoprecipitation with anti-HA magnetic beads in co-transfected HEK 293T cells was performed, SDS-PAGE analysis was then conducuted by using Flag rabbit polyclonal antibody. Results also revealed that Flag-BACE1 band was discovered in the pull-down complex. In conclusion, these results demonstrated that BACE1 interact with protein USP10.

**3. The difference analysis for demographics and clinical variables between AD and HC cohorts**

| Table S2 The result of VIF | | | |
| --- | --- | --- | --- |
|  | GVIF | Df | GVIF^(1/(2*Df)) |
| Sex | 1.475989 | 1 | 1.214903 |
| Age | 1.580281 | 1 | 1.257092 |
| PTEDUCAT | 1.297098 | 1 | 1.138902 |
| PTRACCAT | 2.630474 | 3 | 1.174913 |
| PTMARRY | 1.855065 | 3 | 1.108477 |
| APOE4 | 1.29287 | 2 | 1.066323 |
| MMSE | 2.082728 | 1 | 1.443166 |

| Table S3. Comparisons of demographics and clinical variables between AD and HC cohorts | | | | |
| --- | --- | --- | --- | --- |
|  |  | AD | HC | P-value |
| No. of participants, n (%) |  | 175 (45%) | 214 (55%) |  |
| Sex, n (%) | M | 93 (53%) | 115 (54%) | 0.907 |
|  | F | 82 (47%) | 99 (46%) |  |
| Age, Median (IQR) |  | 75.8(70.85, 81.10) | 75.5(72.12, 78.38) | 0.815 |
| Race, n (%) | Asian | 2 (1) | 2 (1) | 0.357 |
|  | Black | 8 (5) | 15 (7) |  |
|  | More than one | 2 (1) | 0 (0) |  |
|  | White | 163 (93) | 197 (92) |  |
| APOE4, n (%) | 0 | 58 (33) | 156 (73) | ＜0.001 |
|  | 1 | 85 (49) | 53 (25) |  |
|  | 2 | 32 (18) | 5 (2) |  |

Note: APOE4 was coded as the number of epsilon 4 alleles (0, 1, or 2).

**4.The association analysis between the candidated genes on the hippocampal subfields volume and clinical scales**

Table S4 Results of the difference test for the effects of MRGPRX3 gene(11.18159668.T.C) polymorphism on the hippocampal subfields’ volume

Table S5 The results of the difference test for the effects of MRGPRX3 gene(11.18159668.T.C) heterozygous variants on hippocampal subfields’ volume

Table S6 The results of the difference test for the effects of MRGPRX3 gene(11.18159668.T.C) homogenous variants on hippocampal subfields’ volume

Table S7 Results of the difference test for the effects of MRGPRX3 gene (11.18159669.G.A) polymorphism on the hippocampal subfields’ volume

Table S8 The results of the difference test for the effects of MRGPRX3 gene (11.18159669.G.A) heterozygous variants on hippocampal subfields’ volume

Table S9 The results of the difference test for the effects of MRGPRX3 gene (11.18159669.G.A) homogenous variants on hippocampal subfields’ volume

Table S10 Results of the difference test for the effects of NDUFA11 gene(19.5893058.G.A) polymorphism on the hippocampal subfields’ volume

Table S11 The results of the difference test for the effects of NDUFA11 gene(19.5893058.G.A) heterozygous variants on hippocampal subfields’ volume

Table S12 The results of the difference test for the effects of NDUFA11 gene(19.5893058.G.A) homogenous variants on hippocampal subfields’ volume

Table S13 Results of the difference test for the effects of SEPT9 gene(17.75401190.G.A) polymorphism on the hippocampal subfields’ volume

Table S14 The results of the difference test for the effects of SEPT9 gene(17.75401190.G.A) heterozygous variants on hippocampal subfields’ volume

Table S15 The results of the difference test for the effects of SEPT9 gene(17.75401190.G.A) homogenous variants on hippocampal subfields’ volume

Table S16 Results of the difference test for the effects of TRPV1 gene(17.3486702.G.A) polymorphism on the hippocampal subfields’ volume

Table S17 The results of the difference test for the effects of TRPV1 gene(17.3486702.G.A) heterozygous variants on hippocampal subfields’ volume

Table S18 The results of the difference test for the effects of TRPV1 gene(17.3486702.G.A) homogenous variants on hippocampal subfields’ volume

Table S19 Results of the difference test for the effects of USP10 gene(16.84778694.T.C) polymorphism on the hippocampal subfields’ volume

Table S20 The results of the difference test for the effects of USP10 gene(16.84778694.T.C) heterozygous variants on hippocampal subfields’ volume

Table S21 The results of the difference test for the effects of USP10 gene(16.84778694.T.C) homogenous variants on hippocampal subfields’ volume
